# Left Atrial Volumetric and Deformation Analysis in Adult Patients with Dextro-Transposition of the Great Arteries (Insights from the CSONGRAD Registry and MAGYAR-Path Study)

**DOI:** 10.3390/jcm9020463

**Published:** 2020-02-07

**Authors:** Attila Nemes, Gergely Rácz, Árpád Kormányos, Péter Domsik, Anita Kalapos, Nándor Gyenes, Nóra Ambrus, Gábor Bogáts, István Hartyánszky, Kálmán Havasi

**Affiliations:** 2nd Department of Medicine and Cardiology Centre, Medical Faculty, Albert Szent-Györgyi Clinical Center, University of Szeged, H-6725 Szeged, Hungary; racz.gergely@med.u-szeged.hu (G.R.); kormanyos.arpad@med.u-szeged.hu (Á.K.); domsik.peter@med.u-szeged.hu (P.D.); kalapos.anita@med.u-szeged.hu (A.K.); gyenes.nandor@med.u-szeged.hu (N.G.); ambrus.nora@med.u-szeged.hu (N.A.); bogats.gabor@med.u-szeged.hu (G.B.); hartyanszky.istvan@med.u-szeged.hu (I.H.); havasi.kalman@med.u-szeged.hu (K.H.)

**Keywords:** left atrium, function, two-dimensional, three-dimensional, echocardiography, speckle-tracking, transposition of the great arteries

## Abstract

Background: In complete or dextro-transposition of the great arteries (dTGA), the aorta and the pulmonary artery are transposed. The present study was designed to examine dTGA-associated left atrial (LA) volumetric and functional abnormalities in adult patients late after repair and to compare their results to those of healthy controls. Methods: The present study consisted of 15 dTGA patients (30.3 ± 8.1 years, 9 males), the patients had Mustard (*n* = 8) or Senning (*n* = 7) procedure performed. Their results were compared to those of 36 age- and gender-matched healthy subjects (28.7 ± 1.5 years, 24 males). Results: Increased maximum LA volume and reduced LA emptying fractions respecting the cardiac cycle could be demonstrated in our dTGA patients. LA stroke volumes representing all LA functions were significantly reduced. Peak LA circumferential, longitudinal, and area strains and LA circumferential, longitudinal, and area strains measured at atrial contraction were reduced in our dTGA patients. Most LA strains were reduced in patients having Mustard surgery compared to controls and patients undergoing Senning operation. Conclusions: Significant LA volumetric and functional abnormalities could be demonstrated in adult patients with dTGA late after repair. Senning procedure seems to have more beneficial long-term effects on LA volumetric and functional features as compared to the Mustard procedure.

## 1. Introduction

Complete or dextro-transposition of the great arteries (dTGA) is a rare cyanotic congenital heart disease (CHD) representing 5–7% of all CHDs. dTGA affects males in 60–70% [1,2]. In dTGA, the aorta arises from a morphological right ventricle (RV), while the pulmonary artery arises from a morphological left ventricle (LV) leading to parallel running systemic and pulmonary circulations. Due to this special situation, there has to be a communication between the systemic and pulmonary circulation, such as an atrial or ventricular septal defect, or patent ductus arteriosus, allowing the systemic deoxygenated blood to enter the pulmonary circulation. Until the 1990s, the most often used reconstructions were atrial-switch operations, during which an atrial baffle was created. In the case of the Senning procedure, autologous tissue is used to create this baffle to direct the venous return to the contralateral atrioventricular valve and ventricle, while in case of the Mustard procedure, a synthetic material is used for the baffle [3,4].

Three-dimensional (3D) speckle-tracking echocardiography (3DSTE) can be used to perform simultaneous 3D volumetric and strain assessment of different heart chambers, including the left atrium (LA) [5,6,7,8]. 3DSTE allows detailed analysis of reservoir, conduit, and booster pump LA functions at the same time using the same digitally stored 3D echocardiographic datasets [5,8]. Although there is an increasing number of adult dTGA patients who has survived one of the atrial-switch operations during childhood, little is known about their echocardiographic features. Therefore, the present study was designed to examine dTGA-associated LA volumetric and functional abnormalities in adult operated patients and to compare the results with those of healthy controls. Moreover, it was tested whether Senning or Mustard operation is associated with more beneficial LA volumetric and functional properties.

## 2. Materials and Methods

### 2.1. Patient Population

Data of dTGA patients from the Registry of C(S)ONGenital cardiac Disease patients at the University of Szeged (CSONGRAD Registry) were used in this study. This registry contains data of more than 3000 CHD patients [9]. The dTGA patients examined in this study were treated and/or operated on at the Department of Pediatrics, Department of Heart Surgery, and 2nd Department of Medicine and Cardiology Center at the University of Szeged, Hungary between 1961 and 2019. The present study consisted on 15 dTGA patients from this registry, the patients had Mustard (*n* = 8) or Senning (*n* = 7) procedures performed. Complete two-dimensional Doppler echocardiography and 3DSTE were performed in all cases (Table 1). The results of the dTGA patients were compared to those of 36 age- and gender-matched healthy subjects. All dTGA patients and controls were in sinus rhythm. The presented work is part of the MAGYAR-Path Study (Motion Analysis of the heart and Great vessels bYthree-dimensionAl speckle-tRacking echocardiography in Pathological cases), which aimed to assess diagnostic and prognostic significance of 3DSTE-derived parameters even in different kinds of CHD. The study was approved by the institutional human research committee at the Medical Faculty of the University of Szeged (project identification code: 71/2011, date of approval for prolongation: 25/3/2019). The study complied with the 1975 Declaration of Helsinki and informed consent was obtained from each patient and healthy subject.

### 2.2. Two-Dimensional Doppler Echocardiography

All dTGA patients and healthy subjects were in the left lateral decubitus position during standard echocardiographic examination using a commercially available Toshiba Artida^TM^ echocardiography equipment (Toshiba Medical Systems, Tokyo, Japan). During measurements, a PST-30SBP phased-array transducer was used for quantification of LA and LV dimensions according to the guidelines [10]. The presence of valvular regurgitations and stenoses were excluded by Doppler echocardiography. Transmitral E and A flow velocities and their ratio were calculated by pulsed Doppler.

### 2.3. Three-Dimensional Speckle-Tracking Echocardiography

**Figure 1:**
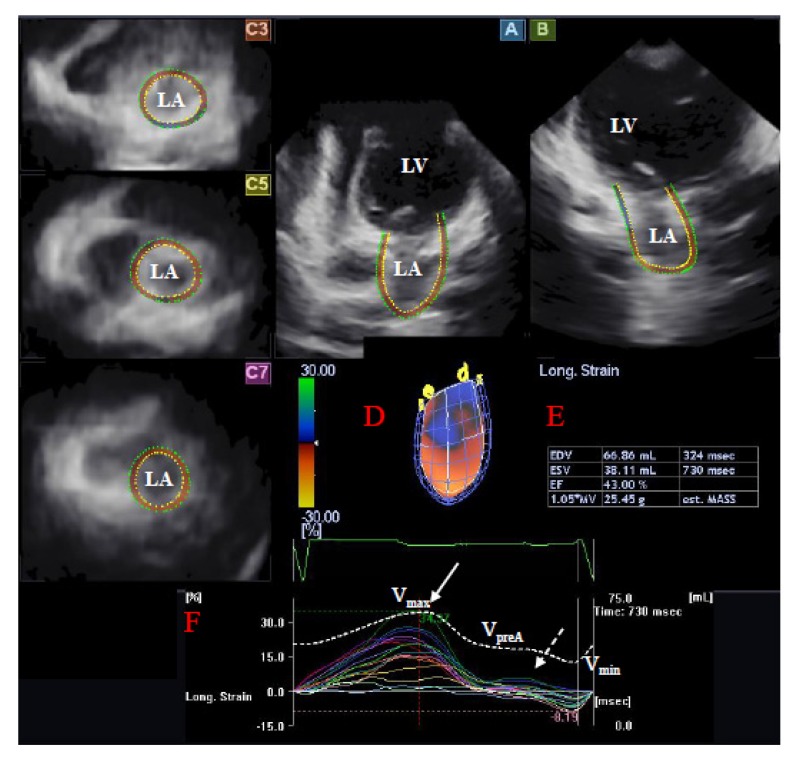
Three-dimensional (3D) speckle-tracking echocardiographic left atrial (LA) analysis using a full-volume dataset in an operated patient with dextro-transposition of the great arteries is presented: (**A**) apical four-chamber view, (**B**) apical two-chamber view, (**C3**) short-axis view at basal, (**C5**) mid- and (**C7**) superior left atrial level. The 3D model of the LA (**D**) and LA volumetric data (**E**) are also presented. Colored curves represent time–LA segmental strains, while dashed curve represents LA volume changes during the cardiac cycle with maximum (V_max_), minimum (V_min_) LA volumes, and LA volume before atrial contraction (V_preA_) (**F**). White arrow represents peak LA strain, while dashed arrow represents LA strain at atrial contraction (**F**). LA = left atrium, LV = left ventricle, V_max_ = maximum LA volume, V_min_ = minimum LA volume, V_preA_ = LA volume before atrial contraction.

Using the same echocardiography equipment, echocardiographic 3D data acquisitions were performed from the apical window using a 1–4 MHz PST-25SX matrix phased-array transducer (Toshiba Medical Systems, Tokyo, Japan) [5,6,7]. Full volume 3D datasets were created from electrocardiographically gated 6 wedge-shaped subvolumes. LA quantification was performed using the 3D Wall Motion Tracking software version 2.7 (Toshiba Medical Systems, Tokyo, Japan). Each dataset was automatically analyzed in apical two-(AP2CH) and four-chamber (AP4CH) views and three short-axis views at different (basal, midatrial, and superior) levels of the LA. The edges of the mitral annulus (MA) and the endocardial side of the superior LA region were defined by the examiner using markers following gain, magnitude etc. optimizations (Figure 1). The endocardial LA surface was then automatically reconstructed and a 3D model of the LA was performed.

### 2.4. Left Atrial 3DSTE-Derived Volumetric Measurements

Using the above mentioned 3D LA cast, the following volumetric parameters were calculated [8,10,11]:

V_max_—end-systolic LA volume defined as the largest LA volume before mitral valve opening,

V_preA_—early diastolic LA volume before atrial contraction at time of P wave on electrocardiogram,

V_min_—late diastolic LA volume defined as the smallest LA volume before mitral valve closure.

From these LA volumetric data, the following LA volume-based functional properties were assessed in order to evaluate systolic reservoir, early diastolic conduit and late diastolic active contraction phases of LA function:

#### 2.4.1. Reservoir Function

TASV—total atrial stroke volume defined as V_max_−V_min_.

TAEF—total atrial emptying fraction defined as TASV/V_max_ × 100. 

#### 2.4.2. Conduit Function

PASV—passive atrial stroke volume defined as V_max_–V_preA_

PAEF—passive atrial emptying fraction defined as PASV/V_max_ × 100.

#### 2.4.3. Active Contraction

AASV—active atrial stroke volume defined as V_preA_−V_min_. 

AAEF—active atrial emptying fraction defined as AASV/V_preA_ × 100.

### 2.5. Left Atrial 3DSTE-Derived Strain Measurements

Unidimensional/unidirectional radial (RS), longitudinal (LS), and circumferential (CS) LA strains and multidimensional/multidirectional/complex area (AS) and 3D (3DS) LA strains were also calculated using the same 3D LA model. To characterize LA reservoir and booster pump functions, global and mean segmental peak strains and strains at atrial contraction were calculated for each patient [8]. Regional apical, midatrial, and basal strains were calculated from segmental strains. LV segmentation model for the LA was used during the assessments. 

### 2.6. Statistical Analysis

All continuous variables are presented as mean values ± standard deviation, while categorical data are summarized as frequencies and percentages. Comparisons were performed with Student *t* test, χ^2^ test, and Fisher’s exact test, when appropriate. Statistical significance was defined if two-tailed *p* value proved to be less than 0.05. Data analysis was carried out using Medcalc software version 13.1.2.0. (MedCalc, Mariakerke, Belgium). 

## 3. Results

### 3.1. Clinical Data

Clinical and two-dimensional echocardiographic data are presented in Table 1. dTGA was associated with atrial septal defect in 2 patients, ventricular septal defect was found in 4 patients, patent ductus arteriosus was present in 5 patients. None of the healthy subjects showed mitral or tricuspid regurgitations, while 2 dTGA patients had grade 1 mitral regurgitation, grade 2–4 mitral regurgitation did not occur. Similarly, 5, 6, 2 dTGA patients had grade 1, 2, or 3–4 tricuspid regurgitation, respectively. None of the healthy controls had significant valvular stenoses. The mean age at the first procedures was 1.4 ± 1.0 years. The mean period between the procedure and the 3DSTE was 29.3 ± 7.9 years.

### 3.2. 3DSTE-Derived LA Volumes and Volume-Based Functional Properties

Increased maximum LA volume and reduced LA emptying fractions respecting the cardiac cycle could be demonstrated in dTGA patients compared to controls. LA stroke volumes featuring all LA functions showed significant reductions. Mustard-operated patients had mostly tendentiously lower stroke volumes and emptying fractions respecting the cardiac cycle compared to those of Senning-operated patients (Table 2).

### 3.3. 3DSTE-Derived LA Peak Strain Parameters

LA global and mean segmental peak LA-RS, LA-CS, LA-LS, and LA-AS proved to be significantly reduced in dTGA patients compared to those of controls. Most peak LA strains were lower in Mustard-operated patients as compared to the peak LA strains of controls and Senning-operated subjects (Table 3). Regional peak LA strains of dTGA patients and controls are presented in Table 4.

### 3.4. 3DSTE-Derived LA Strain Parameters at Atrial Contraction

Similarly to peak LA strains, reduced global and mean segmental LA-CS, LA-AS and reduced mean segmental LA-LS at atrial contraction could be demonstrated in dTGA patients as compared to those of controls (Table 5). Most LA strains measured at atrial contraction proved to be worse in Mustard-operated patients compared to those of Senning-operated cases. Regional LA strains measured at atrial contraction in dTGA patients and controls are presented in Table 6.

## 4. Discussion

It is an important clinical finding that due to improved medical management, more and more dTGA patients appear in the routine clinical practice. However, limited number of studies is available regarding their clinical, morphological, and functional status, therefore each work demonstrating new information about this patient population could help us understand dTGA more and to improve management of adult patients with dTGA having a surgery in the past medical history. Mustard and Senning atrial-switch procedures were the preferred surgeries for dTGA until the 1990s, when arterial switch operation became the preferred technique [12,13]. 

The LA has an important role in the pumping function of the heart. Conventionally, three phases of LA function are differentiated: it serves as a reservoir, when pulmonary venous return is stored in the LA during LV contraction and isovolumetric relaxation. In early diastole, it serves as a conduit, when blood is passively transferred from the LA into the LV (passive emptying), while in late diastole, it serves as a booster pump, when active contraction of the LA is seen (active emptying) [14]. 

3DSTE can be used for detailed assessment of all 3 LA functional phases by volumetric and strain analysis using the same 3D LA cast respecting the cardiac cycle [15]. Volumes, volume-based functional properties, and strain parameters could be calculated for reservoir, conduit, and booster pump LA functions. LA strains are important features of LA contractility. It could be calculated for different directions like radial, longitudinal, and circumferential strains (and their combination) making characterization of LA contractility more accurate and detailed. This characterization of LA function could not be performed with previous conventional echocardiographic parameters [15].

In a recent review, specific disease-related LA abnormalities could be demonstrated in several disorders [8]. In the present study, significant LA volumetric and strain abnormalities could be detected affecting all phases of LA function in dTGA patients. Based on the results, although a relatively small number of patients were examined, Senning procedure seemed to have more beneficial long-term effects on LA volumetric and functional properties as compared to those measured in case of Mustard procedure.

To the best of the authors’ knowledge, this is the first time for detailed 3DSTE-derived characterization of LA volumetric abnormalities and dysfunction in operated adult dTGA patients. Franzoso et al. assessed atrial function in patients after atrial repair in comparison to patients after arterial switch repair and compared the data to controls, magnetic resonance imaging was used in this study [16]. According to their results, abnormal atrial function could be detected in all patients regardless of the type of the procedure. The cyclic volume changes (atrial filling and emptying) were found to be reduced when compared with the changes seen in case of normal subjects. In conclusion, the function of the atria was impaired, and the atria were unable to convert continuous venous flow into a pulsatile flow towards the ventricles. The function of the atrium forwarding pulmonary venous flow into the systemic right ventricle was impaired the most after atrial switch operation. According to these contradictory results, further studies are warranted to assess LA and right atrial (RA) volumes and function respecting the cardiac cycle in dTGA following an atrial switch procedure.

### Limitation Section

Several important limitations have arisen during the presented study as listed below (1) 3DSTE is associated with limited spatial and temporal resolution, which could severely affect the results. (2) 3DSTE measurements could be performed only in sinus rhythm. Measurements in atrial fibrillation are not possible. (3) The shape and localization of the baffle could also have effects on the calculated parameters, which could affect atrial volumetric and functional analyses. (4) Strains of regions not performing contraction (like synthetic material) proved to be 0 during measurements. This could partially explain low strain values of Mustard-operated patients. (5) The present study did not aim to validate 3DSTE-derived parameters due to their validated nature. (6) Only LA variables were measured, volumetric and strain parameters of the RV, RA and LV were not calculated in the present study. (7) Although the use of LA specific segmentation model would have been better, the well-known LV segmentation model was used during LA evaluations.

## 5. Conclusions

Significant LA volumetric and functional abnormalities could be demonstrated in adult patients with dTGA late after repair. Senning procedure seems to have more beneficial long-term effects on LA volumetric and functional features as compared to the Mustard procedure.

## Figures and Tables

**Table 1:** Clinical and two-dimensional echocardiographic parameters of patients with dextro-transposition of the great arteries late after operation and those of controls.

	Controls (*n* = 36)	dTGA Patients (*n* = 15)	*p* Value
**Risk Factors**			
Age (years)	28.7 ± 1.5	30.3 ± 8.1	0.25
Male gender (%)	24 (67)	9 (60)	0.78
Hypertension (%)	0 (0)	4 (21)	0.01
Hypercholesterolemia (%)	0 (0)	0 (0)	1
Diabetes mellitus (%)	0 (0)	0 (0)	1
**Two-Dimensional Echocardiography**			
LA diameter (mm)	37.8 ± 3.9	34.6 ± 6.0	0.08
LV end-diastolic diameter (mm)	46.9 ± 3.3	46.8 ± 4.3	0.91
LV end-diastolic volume (mL)	96.4 ± 22.4	108.7 ± 15.0	0.18
LV end-systolic diameter (mm)	31.5 ± 2.9	30.0 ± 4.0	0.23
LV end-systolic volume (mL)	35.8 ± 7.9	38.3 ± 10.0	0.47
Interventricular septum (mm)	9.0 ± 1.3	10.4 ± 2.5	0.02
LV posterior wall (mm)	9.3 ± 1.7	9.6 ± 1.6	0.62
LV ejection fraction (%)	64.0 ± 3.3	63.7 ± 5.6	0.80

**Abbreviations:** dTGA = dextro-transposition of the great arteries, LA = left atrium, LV = left ventricle.

**Table 2:** Comparison of three-dimensional speckle-tracking echocardiography-derived volumetric left atrial parameters of patients with dextro-transposition of the great arteries and those of controls.

	Controls (*n* = 36)	dTGA Patients (*n* = 15)	Senning-Operated dTGA Patients (*n* = 7)	Mustard-Operated dTGA Patients (*n* = 8)
**Calculated Volumes (mL)**				
V_max_(mL)	42.8 ± 14.8	35.8 ± 22.7 *	37.5 ± 17.9	35.5 ± 29.4 *
V_min_(mL)	20.7 ± 8.4	23.9 ± 18.2	22.5 ± 9.1	26.4 ± 25.9
V_preA_(mL)	30.6 ± 12.9	28.7 ± 19.8	27.5 ± 10.9	31.4 ± 27.6
**Stroke Volumes (mL)**				
TASV (mL)	22.0 ± 10.0	11.9 ± 8.0 *	15.0 ± 10.1	9.1 ± 4.9 *
PASV (mL)	12.1 ± 5.9	7.2 ± 5.7 *	10.0 ± 7.2	4.1 ± 2.0 *
AASV (mL)	9.9 ± 8.1	4.8 ±3.2 *	5.0 ±3.6	5.0 ± 3.3
**Emptying Fractions (%)**				
TAEF (%)	51.0 ± 11.5	33.7 ± 11.2 *	37.5 ± 9.6 *	29.0 ± 12.3 *
PAEF (%)	29.0 ± 12.0	19.4 ± 8.4 *	24.0 ± 7.3	13.1 ± 3.9 *^,†^
AAEF (%)	29.9 ± 15.5	17.8 ± 9.9 *	18.0 ± 8.6 *	18.5 ± 12.2 *

* *p* < 0.05 vs. Controls; † *p* < 0.05 vs. Senning-operated dTGA patients; Abbreviations: dTGA = dextro-transposition of the great arteries, V_max_ = maximum left atrial volume, V_min_ = minimum left atrial volume, V_preA_ = left atrial volume before atrial contraction, TASV = total atrial stroke volume, TAEF = total atrial emptying fraction, AASV = active atrial stroke volume, AAEF = active atrial emptying fraction, PASV = passive atrial stroke volume, PAEF = passive atrial emptying fraction.

**Table 3:** Comparison of three-dimensional speckle-tracking echocardiography-derived peak global and mean segmental left atrial strains of patients with dextro-transposition of the great arteries and those of controls.

	Controls (*n* = 36)	dTGA Patients (*n* = 15)	Senning-Operated dTGA Patients (*n* = 7)	Mustard-Operated dTGA Patients (*n* = 8)
**Global Strains**				
**Radial (%)**	−13.9 ± 7.1	−8.9 ± 7.3 *	−12.8 ± 8.3	−6.2 ± 4.0 *
**Circumferential (%)**	33.1 ± 14.3	11.0 ± 9.4 *	17.1 ± 8.3 *	5.8 ± 7.4 *^,†^
**Longitudinal (%)**	23.9 ± 8.1	12.6 ± 7.9 *	17.3 ± 7.5	6.7 ± 3.1 *^,†^
**3D (%)**	−6.2 ± 5.2	−5.8 ± 4.6	−8.0 ± 5.3	−4.4 ± 2.6
**Area (%)**	64.4 ± 26.4	22.7 ± 17.0 *	34.3 ± 15.7 *	11.1 ± 10.6 *^,†^
**Mean Segmental Strains**				
**Radial (%)**	−19.4 ± 6.7	−13.9 ± 7.1 *	−16.8 ± 8.1	−12.5 ± 5.1 *
**Circumferential (%)**	37.4 ± 13.7	15.5 ± 9.5 *	21.7 ± 9.5 *	9.8 ± 6.0 *^,†^
**Longitudinal (%)**	28.1 ± 7.8	15.4 ± 7.3 *	19.1 ± 7.7 *	10.6 ± 3.3 *^,†^
**3D (%)**	−12.4 ± 5.2	−10.0 ± 5.6	−12.6 ± 6.2	−8.7 ± 3.4 *
**Area (%)**	71.2 ± 26.1	28.2 ± 16.5 *	39.2 ± 16.9 *	17.6 ± 8.7 *^,†^

* *p* < 0.05 vs. Controls; † *p* < 0.05 vs. Senning-operated dTGA patients; Abbreviations: dTGA = dextro-transposition of the great arteries, 3D = three-dimensional.

**Table 4:** Comparison of three-dimensional speckle-tracking echocardiography-derived peak regional left atrial strains of patients with dextro-transposition of the great arteries and those of controls.

	Controls (*n* = 36)	dTGA Patients (*n* = 15)	Senning-Operated dTGA Patients (*n* = 7)	Mustard-Operated dTGA Patients (*n* = 8)
**RS _basal_ (%)**	−19.0 ± 8.3	−14.8 ± 8.9	−17.0 ± 10.1	−14.3 ± 7.4 *
**RS_midatrial_ (%)**	−19.4 ± 7.5	−13.6 ± 7.3 *	−14.7 ± 8.4	−13.4 ± 6.7
**RS _superior_ (%)**	−17.1 ± 10.8	−13.2 ± 10.7	−19.4 ± 12.1	−8.3 ± 5.7
**CS _basal_ (%)**	40.1 ± 14.6	13.8 ± 8.8 *	20.7 ± 7.2 *	8.1 ± 5.2 *^,†^
**CS_midatrial_ (%)**	31.2 ± 12.7	12.2 ± 9.6 *	17.9 ±10.7 *	7.2 ± 5.3 *^,†^
**CS _superior_ (%)**	40.2 ± 25.0	22.9 ± 15.7 *	29.0 ± 17.2 *	16.5 ± 13.5 *^,†^
**LS _basal_ (%)**	19.1 ± 9.1	16.6 ± 9.3	19.1 ± 7.0	11.6 ± 8.2 *
**LS_midatrial_ (%)**	35.7 ± 11.0	15.6 ± 8.8 *	21.0 ± 9.3 *	9.9 ± 4.8 *^,†^
**LS _superior_ (%)**	27.8 ± 16.5	13.3 ± 8.9 *	16.3 ± 10.5	9.9 ± 6.9 *
**3DS _basal_ (%)**	−12.4 ± 6.2	−11.0 ± 7.1	−13.1 ± 8.1	−10.1 ± 5.8
**3DS_midatrial_ (%)**	−11.3 ± 5.5	−8.6 ± 5.2	−9.7 ± 5.6	−8.6 ± 4.5
**3DS _superior_ (%)**	−10.8 ± 8.1	−10.6 ± 10.1	−16.2 ± 11.9	−6.5 ± 5.0
**AS _basal_ (%)**	58.1 ± 23.2	23.1 ± 13.1 *	32.3 ± 11.3 *	13.8 ± 8.5 *^,†^
**AS_midatrial_ (%)**	71.7 ± 26.5	24.9 ± 19.2 *	37.0 ± 21.1*	13.4 ± 9.5 *^,†^
**AS _superior_ (%)**	85.7 ± 62.6	40.6 ± 29.6 *	52.6 ± 33.3	29.4 ± 24.7 *

* *p* < 0.05 vs. Controls; † *p* < 0.05 vs. Senning-operated dTGA patients; Abbreviations: dTGA = dextro-transposition of the great arteries, RS = radial strain, CS = circumferential strain, LS = longitudinal strain, 3DS = three-dimensional strain, AS = area strain.

**Table 5:** Comparison of three-dimensional speckle-tracking echocardiography-derived regional left atrial strains at atrial contraction of patients with dextro-transposition of the great arteries and those of controls.

	Controls (*n* = 36)	dTGA Patients (*n* = 15)	Senning-Operated dTGA Patients (*n* = 7)	Mustard-Operated dTGA Patients (*n* = 8)
**Global Strains**				
**Radial (%)**	−4.9 ± 6.6	−3.6 ± 4.8	−4.3 ± 5.0.	−3.8 ± 4.6
**Circumferential (%)**	13.6 ± 8.3	4.1 ± 8.8 *	9.5 ± 10.1	0.7 ± 4.0 *^,†^
**Longitudinal (%)**	7.6 ± 6.2	5.5 ± 6.2	5.9 ± 7.2	3.4 ± 3.3
**3D (%)**	−3.0 ± 5.7	−1.8 ± 5.4	−3.6 ± 6.5	−1.1 ± 3.1
**Area (%)**	23.5 ± 17.6	9.1 ± 16.6 *	16.7 ± 21.2	1.2 ± 7.3 *
**Mean Segmental Strains**				
**Radial (%)**	−8.0 ± 5.4	−6.1 ± 4.4	−6.4 ± 5.4	−6.8 ± 3.2
**Circumferential (%)**	16.4 ± 8.5	6.1 ± 7.9 *	10.0 ± 9.6	2.3 ± 4.1 *
**Longitudinal (%)**	9.5 ± 5.4	6.7 ± 5.0 *	7.9 ± 5.3	4.0 ± 2.1 *
**3D (%)**	−5.4 ± 5.1	−4.0 ± 4.2	−4.6 ± 5.1	−4.4 ± 2.5
**Area (%)**	27.6 ± 13.6	10.6 ± 12.1 *	16.6 ± 14.4	4.1 ± 6.5 *

* *p* < 0.05 vs. Controls; † *p* < 0.05 vs. Senning-operated dTGA patients; Abbreviations: dTGA = dextro-transposition of the great arteries, 3D = three-dimensional.

**Table 6:** Comparison of three-dimensional speckle-tracking echocardiography-derived regional left atrial strains at atrial contraction of patients with dextro-transposition of the great arteries and those of controls.

	Controls (*n* = 36)	dTGA Patients (*n* = 15)	Senning-Operated dTGA Patients (*n* = 7)	Mustard-Operated dTGA Patients (*n* = 8)
**RS _basal_ (%)**	−8.1 ± 6.2	−6.2 ± 5.8	−5.5 ± 6.2	−8.1 ± 4.9
**RS_midatrial_ (%)**	−8.4 ± 6.3	−5.5 ± 4.2	−4.9 ± 3.0 *	−6.8 ± 5.2
**RS _superior_ (%)**	−7.4 ± 8.3	−6.9 ± 8.8	−9.8 ± 11.3	−4.9 ± 5.3
**CS _basal_ (%)**	17.9 ± 10.6	4.7 ± 6.3 *	7.7 ± 7.5 *	2.2 ± 3.9 *
**CS_midatrial_ (%)**	12.9 ± 8.2	5.5 ± 7.8 *	9.4 ± 9.4	1.8 ± 4.5 *
**CS _superior_ (%)**	17.3 ± 14.9	9.0 ± 11.4 *	14.4 ± 14.3	3.2 ± 5.2 *
**LS _basal_ (%)**	6.7 ± 5.6	6.9 ± 6.2	5.4 ± 3.7	5.9 ± 4.4
**LS_midatrial_ (%)**	11.1 ± 7.1	6.5 ± 6.2 *	8.4 ± 7.6	3.5 ± 3.0 *
**LS _superior_ (%)**	11.3 ± 10.9	6.5 ± 8.7	10.9 ± 10.5	1.9 ± 4.2 *
**3DS _basal_ (%)**	−5.5 ± 6.5	−4.4 ± 5.7	−4.7 ± 6.6	−5.5 ± 4.0
**3DS_midatrial_ (%)**	−5.5 ± 5.9	−3.3 ± 3.6	−3.2 ± 3.2	−4.1 ± 3.9
**3DS _superior_ (%)**	−4.9 ± 7.6	−4.5 ± 7.3	−6.7 ± 9.3	−3.3 ± 4.9
**AS _basal_ (%)**	22.7 ± 12.5	6.9 ± 7.1 *	9.1 ± 7.5 *	3.5 ± 5.4 *
**AS_midatrial_ (%)**	27.3 ± 15.5	11.8 ± 13.9 *	19.1 ± 16.4	4.2 ± 7.0 *
**AS _superior_ (%)**	35.6 ± 32.3	14.4 ± 20.3 *	24.0 ± 25.3	4.7 ± 10.2 *

* *p* < 0.05 vs. Controls; Abbreviations: dTGA = dextro-transposition of the great arteries, RS = radial strain, CS = circumferential strain, LS = longitudinal strain, 3DS = three-dimensional strain, AS = area strain.
